# Supplementary material for: Pathophysiological Significance of Neutrophilic Transfer RNA-Derived Small RNAs in Asymptomatic Moyamoya Disease
Source: Cells. 2021 May 1;10(5):1086. doi: 10.3390/cells10051086 (PMC8147334; doi:10.3390/cells10051086)
Supplement: Supplementary file 1 [file cells-10-01086-s001.zip › cells-1149161 3rd supplementary materials/Data Supplements/Data Supplement V.zip!/Data supplement 6-mRNAs targets of tsRNAs/tsRNA-Glu-TTC-010/Diagrams/tRF-Glu-TTC-010_vs_ENST00000314393(ZHX2).pdf]

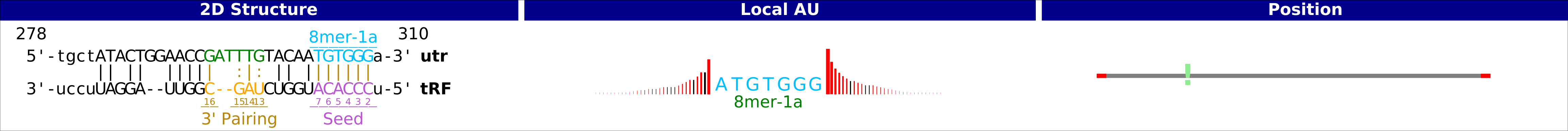

2D Structure
Local AU
Position
278
310
8mer-1a
5'-tgctATACTGGAACCGATTTGTACAATGTGGGa-3' utr
3'-uccuUAGGA--UUGGC--GAUCUGGUACACCCu-5' tRF
16
15 14 13
7 6 5 4 3 2
3' Pairing
Seed
ATGTGGG
8mer-1a
